# Effect of co-morbidities on the development of oral feeding ability in pre-term infants: a retrospective study

**DOI:** 10.1038/srep16603

**Published:** 2015-11-12

**Authors:** Maria Lorella Giannì, Patrizio Sannino, Elena Bezze, Laura Plevani, Nathalie di Cugno, Paola Roggero, Dario Consonni, Fabio Mosca

**Affiliations:** 1 Fondazione I.R.C.C.S. Ca Granda Ospedale Maggiore Policlinico, Neonatal Intensive Care Unit Department of Clinical Science and Community Health, University of Milan, Via Commenda 12, 20122 Milano, Italy; 2 Fondazione I.R.C.C.S. Ca Granda Ospedale Maggiore Policlinico, S.I.T.R.A. Basic Education Sector, Via Francesco Sforza 28, 20122 Milano, Italy; 3 Fondazione IRCCS Ca’ Granda Ospedale Maggiore Policlinico, Epidemiology Unit, Via San Barnaba 8, 20122 Milan, Italy

## Abstract

Pre-term infants frequently experience difficulties in attaining independent oral feeding, thus delaying the achievement of an adequate nutritional status and hospital discharge. The aim of this retrospective, single-centre, observational study was to investigate the effect of co-morbidities on the timing of the achievement of full oral feeding in pre-term infants. The neonatal and feeding data of 84 infants born at a gestational age of <32 weeks were collected, and the effect of co-morbidities on the achievement of feeding independence was evaluated using multiple linear regression analysis. The mean postmenstrual age at the time of the achievement of full oral feeding was 36.7 ± 3.68 weeks (range 33–53) weeks. The multiple linear regression analysis showed that a low birth weight, the occurrence of bronchopulmonary dysplasia, and the need for gastrointestinal surgical procedures were independently associated with a higher postmenstrual age at achievement of full oral feedings.

It is widely acknowledged that pre-term infants frequently experience difficulties in attaining independent oral feeding, thus delaying the achievement of an adequate nutritional status and hospital discharge which, according to the American Academy of Pediatrics, requires the ability to feed exclusively by mouth^1^,^2^,^3^. The development of oral feeding ability in pre-term infants is therefore a primary concern for neonatologists.

Because of the immaturity of their body functions and the occurrence of co-morbidities, pre-term infants generally require a period of enteral feeding before they acquire the coordinated sucking ability that allows sufficient milk intake for growth^4^. Furthermore, early life experiences driven by an unfavourable environment in the neonatal intensive care unit (NICU), which are characterised by exposure to non-physiologic stimuli such as excessive lighting, noise and stressful and painful procedures, can further hinder the post-natal maturation of oral motor skills^5^.

Human milk is the best food for pre-term infants because it provides a number of health benefits^6^, but although it is recommended, the number of very low birth weight infants feeding on human milk at the time of hospital discharge is still limited^7^. It has also been shown that a neonatal intensive care unit environment is particularly challenging with regard to breastfeeding; thus, a large percentage of pre-term infants is offered a bottle before being put to the breast when they are developmentally ready to feed^8^. Indeed, the provision of expressed human milk by bottle may be necessary in the case of maternal-infant separation^8^, and NICUs’ better staffing ratios have been reported to positively affect breastfeeding^9^. Furthermore, infant sucking skills differ between bottle- and breastfeeding^10^, and pre-term infants have been reported to show a weaker and less coordinated suck^11^.

Few studies have investigated the timing and effect of co-morbidities on the achievement of independent oral skills. Jadcherla *et al.*^5^ reported that infants born with a gestational age of <28 weeks showed a significant delay in attaining the transition to oral feeding, but it has also been found that the occurrence of morbidities negatively correlates with postmenstrual age at the time of achieving oral feeding skills^5^,^12^,^13^,^14^.

The aim of this study was to investigate the effect of co-morbidities on the timing of the achievement of full oral feeding in a cohort of pre-term infants and to evaluate their mode of feeding and feeding status at the time of hospital discharge.

## Methods

### Design and setting

This retrospective, single-centre observational study was carried out in a level III neonatal intensive care unit in accordance with the guidelines. The study was approved by the Ethics Committee of Fondazione Istituto di Ricovero e Cura a Carattere Scientifico Cà Granda Ospedale Maggiore Policlinico, and written informed consent was obtained from the parents.

### Sample

All of the consecutive newborns admitted to the authors’ institution between January and 31 December 2012 were screened for eligibility. The inclusion criteria were a gestational age of <32 weeks and Caucasian parentage; the exclusion criteria were known congenital and/or chromosomal diseases, death during hospitalisation, or transfer to another institution.

### Data collection

The neonatal, medical and feeding data from birth to discharge were collected from the patients’ computerised medical charts. The neonatal data recorded were gestational age at birth, birth weight, gender, singleton pregnancies, mode of delivery, 5-minute APGAR score, length of hospitalisation and postmenstrual age (gestational age plus chronological age: PMA) at the time of discharge. Gestational age was based on the date of the last menstrual period and the findings of the first-trimester ultrasonogram. The infants with a birth weight <10^th^ percentile or 10^th^–90^th^ percentiles for gestational age based on Fenton’s growth chart^15^ were classified as being either small for gestational age (SGA) or appropriate for gestational age (AGA), respectively. The medications recorded included pre- and post-natal steroids, surfactant therapy, and inotropic agents.

A record was also made of the occurrence of hemodynamically significant patent ductus arteriosus (PDA), classified based on the McNamara and Sehgal staging system^16^; ≥Bell’s stage III necrotising enterocolitis (NEC)^17^; any condition requiring surgery; ≥stage 3 retinopathy of prematurity (ROP) according to the revised international classification^18^; ≥Papile stage 3 intraventricular haemorrhage (IVH)^19^; periventricular leukomalacia, defined as the combined presence of focal necrosis in the periventricular region and diffuse reactive gliosis in the surrounding white matter^20^; sepsis defined as the presence of a positive blood culture; and mild, moderate or severe bronchopulmonary dysplasia (BPD), defined based on the classification of Jobe and Bancalari^21^.

The feeding data included postmenstrual age at the time of starting minimal enteral feeding (defined as the enteral administration of a milk volume of ≤20 ml/kg/day); postmenstrual age at the time of starting enteral feeding (defined as the enteral administration of a milk volume of >20 ml/kg/day); the duration of enteral feeding (defined as the number of days during which any milk volume was enterally administered); postmenstrual age at the time of starting oral feeding (defined as the consumption of at least 5 ml of milk within 24 hours, directly at the breast or by bottle); and postmenstrual age at the time of achieving full oral feeding (defined as the consumption of all of the milk, at the breast or by bottle). A record was also made of the mode of feeding (directly at the breast, exclusively by bottle, or a mixture of both) and feeding status at discharge, according to the World Health Organisation’s definition of exclusively human milk, any human milk, or exclusively formula^22^.

### Feeding practices

Parenteral nutrition was started immediately after birth; enteral feeding was started within 24 hours of post-natal life using human milk or pre-term formula when human milk was unavailable. When the infants tolerated an enteral intake of >100 ml/kg/day, individually tailored fortification of human milk and/or pre-term formula was started. Oral feeding was started when the infants showed oromotor cues, and feeding progression was carried out based on the infants’ cardiorespiratory stability and gastrointestinal (GI) tolerance. Lactation counselling was provided as described in detail elsewhere^23^. Skin-to-skin contact was promoted provided that the infant was in stable clinical condition. All of the NICU staff encouraged mothers to give drops of their milk during kangaroo care, and mothers were encouraged to breastfeed their infants even for a short time once their clinical condition had stabilised.

### Statistical analysis

The descriptive data of all of the enrolled infants are given as the mean values and standard deviation (SD) or the number of observations and percentages. For the purposes of analysis, the only infant who developed NEC, which required surgery, was pooled with the infants who had any other condition requiring GI surgery. Furthermore, infants developing intraventricular haemorrhage and/or periventricular leukomalacia and those developing retinopathy of prematurity were pooled together and classified as being affected by neurosensory diseases.

The association among comorbidities (yes *vs* no), birth weight, gestational age and PMA at the start of oral feeding and at the time of full oral feeding was assessed using univariate linear regression analysis. A multiple linear regression model was used to identify the determinants of PMA at the time of full oral feeding. To avoid collinearity, we included birth weight rather than gestational age or being SGA as independent variable because it was most closely correlated with PMA at the time of achieving full oral feeding. For the same reason, BPD (yes *vs* no) rather than the administration of postnatal surfactant or steroids was included. GI surgical disease (yes *vs* no), PDA (yes *vs* no), sepsis (yes *vs* no), and neurosensory disease (yes *vs* no) further entered the model as independent variables.

Statistical analyses were made using SPSS (Statistical Package for the Social Sciences) version 12 software (SPSS Inc., Chicago, IL, USA).

## Results

The study involved a total of 84 infants, whose basic characteristics are shown in Table 1.

Their mean length of hospital stay was 66.3 ± 44 days (range 15–242), and their mean postmenstrual age and weight at the time of discharge were 38.2 ± 4.2 weeks (range 33–57) and 2685 ± 746 g (range 1900–5615), respectively.

Minimal enteral feeding was started on day 2.4 ± 3.5 (range 0–20), when their mean PMA was 30.6 ± 1.7 weeks (range 26–35). The mean duration of enteral feeding was 27.2 ± 23.9 days (range 8–150). The mean PMA at the time of starting and achieving full oral feeding was 33.7 ± 1.5 weeks (range 31–43) and 36.7 ± 3.7 weeks (range 33–53), respectively.

At the time of discharge, 56 infants (67%) were exclusively bottle feeding, 27 (32%) were feeding at the breast and by bottle, and only one was being enterally fed; fifteen infants (18%) were fed exclusively human milk, 40 (48%) infants were fed any human milk, and 29 (34%) were exclusively formula fed.

Tables 2 and 3 show the feeding data by the infants’ basal characteristics and the occurrence of co-morbidities. Mean PMA at the start of oral feeding decreased with the increase of birth weight but not so clearly with the increase of gestational age and it increased in infants who developed BPD or sepsis compared with those who did not. PMA at the time of the achievement of full oral feeding significantly decreased with the increase of both gestational age and birth weight, whereas it significantly increased in infants who underwent GI surgery and those who developed sepsis, PDA, BPD or neurosensory diseases compared with those who did not. The mean PMA in both dimensions increased significantly more among the SGA than among the AGA infants (Table 3).

The multiple linear regression analysis (R^2^ = 0.625, p < 0.0001) showed that a low birth weight, the occurrence of BPD, undergoing GI surgery, and possibly having a neurosensory disease were all independently associated with an older PMA at the time of achieving full oral feeding (Table 4).

## Discussion

The findings of this study indicate that birth weight was independently associated with PMA at the time of the achievement of full oral feeding: the lower the birth weight was, the older the PMA was. This finding suggests that a low birth weight *per se* is a risk factor for oral feeding difficulties. Furthermore, the univariate linear regression showed that gestational age had a tendency (p = 0.07) to be negatively correlated with PMA at the time of starting oral feeding and that it negatively correlated at the time of achieving full oral feeding. Indeed, it is acknowledged that the evolution of neurodevelopmental and gut motility is highly dependent on perinatal maturation^5^,^24^,^25^ such that extremely pre-term infants have immature oral motor skills and lack coordinated sucking ability^1^. Moreover, there was a positive correlation with being born SGA which is a known risk factor for a prolonged transition period from starting oral feeding to full oral feeding^26^, and persistent feeding delay during the first nine months of corrected age^27^. It is also necessary to consider the fact that the post-natal maturation of pre-term infants is affected by non-physiological and stressful environmental conditions that can further delay the development of adequate central nervous system function, including the acquisition of the oral motor skills required for feeding success^5^,^28^.

Univariate linear regression analysis showed that the occurrence of BDP and sepsis correlated with an older PMA at the time of starting oral feeding and at the time of transition to full oral feeding. Furthermore, the occurrence of PDA and neurosensory diseases as well as the need for GI surgery were positively correlated with an older PMA at the time of achieving full oral feeding. However, multiple linear regression analysis showed that only the need for GI surgery and the occurrence of BPD independently correlated with PMA at the time of full oral feeding, thus underlining the impact of specific co-morbidities on progression to full oral feeding. In addition, the occurrence of neurosensory diseases showed a tendency to be independently associated with a delay in the attainment of full oral feeding (p = 0.06). It can be speculated that this variable could not reach full statistical significance due to the small number of subjects who developed this specific comorbidity. Not surprisingly, surgery was associated with a longer delay by 3.6 weeks because infants are not normally allowed to be fully orally fed until they have completely recovered. BPD led to a delay of 2 weeks, possibly because it can disrupt the individual rhythms of sucking, swallowing and breathing, which are critical for achieving coordinated suckling^29^. Indeed, pre-term infants with BPD have been reported to need 15–28 days longer than healthy preterm infants to achieve full oral feeding^30^.

The findings of the present study are in accordance with those of previous studies. Jadcherla *et al.*^5^ retrospectively investigated feeding milestones in the progression to oral feeding in 186 pre-term infants and found that full oral feeding was achieved at a PMA of 35 ± 1.5 weeks in the case of infants born at a gestational age of 28–32 weeks but at a PMA of 36.6 ± 2.4 weeks in those born at a gestational age of <28 weeks. Furthermore, their regression analysis showed that infants undergoing prolonged ventilation and/or nasal continuous positive airway pressure therapy and those developing hypotension achieved full oral feeding at a significantly later PMA. Hwang *et al.*^14^ investigated the duration of the period of transition from starting oral feeding to achieving full oral feeding and the medical complications that could negatively interfere with feeding progress in a cohort of 117 pre-term infants; the infants’ mean PMA at the end of the period was 35.1 ± 2.0 weeks. The slight difference between their findings and our own may be at least partially explained by the fact that their infants were heavier at birth (1325 ± 362 *vs* 1262 ± 404 g) and that they included a smaller proportion of infants who were SGA (3.4% *vs* 19%). Furthermore, as with our findings, Hwang *et al.*^14^ found that BPD and NEC were the most important medical complications associated with an older PMA at the time of achieving full oral feeding. White-Traut *et al.* investigated oral feeding progression in 142 stable pre-term infants^31^ and found that it took 7.6 ± 5.6 days to make the transition, with a longer transition correlating negatively with a lower birth weight and positively with greater infant morbidity. Van Nostrand *et al.*^12^ conducted a retrospective study on a large cohort of pre-term infants who were born before the 37^th^ completed week of gestation and reported that the mean PMA at achieving full oral feeding was 36 ± 4/7 weeks, with the oldest PMA occurring in the most premature infants. Consistently with our findings, a delay in the achievement of full oral feeding was associated with the occurrence of specific morbidities, specifically BPD, NEC and severe intraventricular haemorrhage. Dodrill *et al.*^1^ investigated the age at which 472 preterm infants who were born with a gestational age of less than 37 weeks and were admitted to a level II and III nursery over a 12-month period attained full oral feeding. The authors reported that both a low gestational age and a high degree of morbidity correlated with a higher PMA at the attainment of independent oral feeding. Park *et al.*^13^ focused on the impact of comorbidities on the achievement of independent oral feeding in 94 extremely preterm infants who were born before 28 weeks of gestation. These authors found that the achievement of feeding milestones was slower for younger infants and for infants who developed BPD, NEC, PDA and neurologic diseases.

Only one of our infants failed to achieve independent oral feeding and was consequently discharged on enteral feeding. A pre-term infant’s readiness for oral (breast or bottle) feeding is an essential criterion for discharge from an NICU^3^, and a great deal of research has been undertaken to gain further insights into the factors that positively affect its development. A number of authors have investigated the potentially beneficial effects of non-nutritive sucking or oral stimulation, but their findings are conflicting. There is also increasing evidence indicating that oral feeding readiness should be assessed on the basis of acquired developmental and behavioural feeding cues rather than feeding volume and duration^32^. Horner *et al.*^33^ examined the efficacy of an intervention directed to support feeding development in medically complex preterm infants. The authors reported a significant reduction in the number of days needed to attain exclusive oral feeding in the infants who were enrolled in the intervention program compared with infants who had received standard care (10.8 vs 19.3 days, p = 0.01). Indeed, the mean PMA at which the medically complex enrolled infants attained independent oral feeding was 37.8 weeks, which is actually only one to two weeks later than the mean PMA age recorded for preterm infants who are not affected by comorbidities in the present study and in previous ones^34^,^35^.

The American Academy of Pediatrics has recommended that all pre-term infants should receive human milk because of its health benefits^36^; it is thus highly important to promote their direct breastfeeding^37^,^38^. In the present study, we failed to achieve exclusive direct breastfeeding in any infant, whereas 32% were fed human milk both by bottle and at the breast. This result reflects the difficulties encountered in breastfeeding premature infants. Pineda *et al.*^39^ reported that 48% of their 66 extremely pre-term infants were never put to the breast during their hospital stay and found a positive relationship between direct breast-feeding behaviours in a NICU and successful breastfeeding at discharge. The priority of breastfeeding support in NICU was also noted by Maastrup *et al.*^40^, who prospectively investigated PMA at breastfeeding milestones and found that up to 50% of the enrolled extremely pre-term infants were exclusively breastfed at discharge.

The failure to achieve exclusive direct breastfeeding in any infant in the present study may be partly explained by the fact that the setting of the study did not have as must lactation support as described in the study by Maastrup *et al.*^40^. Indeed, the authors speculated that the high percentage of preterm infants who were discharged home and were directly breastfed may reflect the high priority of supporting breastfeeding in NICUs, which is associated with the cultural custom of breastfeeding initiation and is typical of Denmark. In a retrospective study of 88 infants born at a gestational age of <34 weeks, Briere *et al.*^8^ reported that 51% of the 41 preterm infants who were fed exclusively human milk had directly breast fed two days before discharge. However, it must be taken into account that in the study by Briere *et al.*, 46% of the enrolled infants were older, with a gestational age at birth of 32 and 33 weeks, than were the infants enrolled in the present study; thus, the achievement of direct breastfeeding in these infants could have been easier due to their better neurodevelopmental maturation. Furthermore, only infants who had received maternal human milk for longer than 7 days were enrolled, whereas infants with a diagnosis of short bowel syndrome and those who were discharged home still requiring tube feeding were excluded.

In the present study, we reported a lower percentage (18%) of infants fed human milk exclusively than the percentage found in previous studies. Gibertoni *et al.*^41^ reported exclusive human milk feeding in 34% of very low birth weight infants at discharge. It can be speculated that in the present study, some mothers may not have had a clear breastfeeding goal because premature delivery often occurs unexpectedly such that they may not have finalised their infant feeding plans. Davanzo *et al.*^7^ conducted a multicentre study involving 13 Italian NICUs and found that 31% of the preterm infants with a gestational age at birth less than 32 weeks were fed human milk exclusively. However, it must be noted that this figure comprises exclusive human milk feeding, including expressed and donated breast milk. Briere *et al.*^8^ reported that 46% of the enrolled preterm infants were fed human milk exclusively. The relatively high percentage of infants fed human milk exclusively may be due to the particularly strong breastfeeding support that characterised the study’s setting. However, when considering any human milk feeding, 66% of the infants enrolled in the present study were fed any human milk, which is consistent with the figures reported by other authors^7^,^8^,^41^.

This study has the same limitations as any retrospective study. Furthermore, it was not possible to assess some of the variables that are known to affect the development of feeding skills, such as mother-father-infant relationships or the frequency of kangaroo mother care sessions during the hospitalisation of each infant. Nevertheless, the fact that it was a single-centre study means that the achievement of feeding ability was not affected by inconsistent NICU feeding guidelines, and the number of enrolled infants was relatively large.

## Conclusions

Our findings should encourage clinicians and nurses to concentrate on infants born with a low birth weight or who develop BDP and/or medical complications requiring surgery when assessing oral feeding readiness. Further studies are required to develop evidence-based interventions to promote the achievement of feeding independence and direct breastfeeding, which can reduce the duration of hospitalisation and the costs of healthcare.

## Additional Information

**How to cite this article**: Giannì, M. L. *et al.* Effect of co-morbidities on the development of oral feeding ability in pre-term infants: a retrospective study. *Sci. Rep.* **5**, 16603; doi: 10.1038/srep16603 (2015).

## Figures and Tables

**Table 1:** Descriptive data (n = 84).

*Basic characteristics*	mean (SD)
Gestational age (weeks)	29.2 (1.2)
Birth weight (g)	1262 (404)
Apgar score 1 min	6.1 (1.6)
Apgar score 5 min	8.1 (0.8)
	**n (%)**
Caesarean section	81 (96)
Males	39 (46)
Multiple gestation	45 (54)
SGA	16 (19)
***Medications***	**n (%)**
Prenatal steroids	76 (90)
Surfactants	38 (45)
Postnatal steroids	11 (13)
***Medical complications***	**n (%)**
Sepsis	27 (32)
PDA pharmacological treatment	22 (26)
Mild BPD	5 (6)
Moderate BPD	7 (8)
Severe BPD	6 (7)
IVH/PVL	3 (4)
GI surgical procedures	6 (7)
PDA surgery	2 (2)
ROP	7 (8)

SGA: small for gestational age; PDA: patent ductus arteriosus; BPD: bronchopulmonary dysplasia. IVH: intraventricular haemorrhage; PVL: periventricular leukomalacia; GI: gastrointestinal. ROP: retinopathy of prematurity.

**Table 2:** Feeding data by the infants’ basal characteristics and the occurrence of co-morbidities.

	N	PMA at start of oral feeding (weeks)	N	PMA at time of full oral feeding (weeks)
		Mean (SD)		Mean (SD)
AGA	68	33.4 (0.9)	68	36.0 (2.2)
SGA	16	35.0 (2.4)	15	40.0 (6.1)
BPD				
No	66	33.4 (1.0)	66	35.6 (1.6)
Yes	18	34.9 (2.3)	17	41.1 (5.5)
PDA				
No	60	33.5 (1.6)	60	35.8 (2.5)
Yes	24	34.1 (1.1)	23	38.9 (5.01)
Sepsis				
No	57	33.3 (0.9)	57	35.6 (1.6)
Yes	27	34.6 (2.0)	26	39.1 (5.3)
Surgical procedures				
No	78	33.6 (1.5)	77	36.4 (3.2)
Yes	6	34.8 (0.7)	6	40.8 (5.9)
Neurosensory diseases				
No	75	33.6 (1.5)	75	35.8 (2.2)
Yes	9	34.4 (1.3)	8	41.6 (5.9)

PMA: postmenstrual age; SGA: small for gestational age; AGA: appropriate for gestational age; BPD: bronchopulmonary dysplasia; PDA: patent ductus arteriosus.

**Table 3:** Univariate linear regression analysis of the association between infants’ characteristics, co-morbidities and PMA at the start of oral feeding and at the achievement of full oral feeding.

	PMA at the start of oral feeding (weeks)				PMA at the achievement of full oral feeding (weeks)			
	R^2^	β	95% CI	*p*	R^2^	β	95% CI*	*p*
Birth weight (100 g)	0.32	−0.2	−0.3;−0.1	<0.0001	0.43	−0.6	−0.7;−0.4	<0.0001
Gestational age (weeks)	0.03	−0.2	−0.3;0.01	0.07	0.23	−0.9	−1.3;−0.5	<0.0001
Being SGA vs being AGA	0.16	1.6	0.7;2.3	<0.0001	0.18	4.06	2.1;5.9	<0.0001
BPD (yes *vs* no)	0.18	1.5	0.8;2.2	<0.0001	0.38	5.5	3.9;7.1	<0.0001
PDA (yes *vs* no)	0.03	0.6	−0.1;1.3	0.90	0.14	3.1	1.4;4.7	<0.0001
Sepsis (yes *vs* no)	0.17	1.3	0.6;1.9	<0.0001	0.20	3.5	1.9;5.1	<0.0001
GI Surgical procedures (yes *vs* no)	0.04	1.2	−0.1;2.4	0.06	0.10	4.4	1.5;7.3	0.03
Neurosensory diseases (yes *vs* no)	0.03	0.8	−0.2;1.8	0.13	0.32	6.9	4.7;9.1	<0.0001

PMA: postmenstrual age; CI: confidence interval; SGA: small for gestational age; AGA: appropriate for gestational age; BPD: bronchopulmonary dysplasia; PDA: patent ductus arteriosus; GI: gastrointestinal.

**Table 4:** Multiple linear regression analysis of the associations among birth weight, co-morbidities, and PMA at the time of full oral feeding.

	PMA at the achievement of full oral feeding (weeks)		
	β	95% CI	p
Birth weight (100 g)	−0.3	−0.5; −0.2	<0.0001
BPD (yes *vs* no)	2.0	0.2; 3.8	0.03
GI surgical diseases (yes *vs* no)	3.6	1.6; 5.6	0.001
PDA (yes *vs* no)	−0.5	−1.8; 0.8	0.44
Sepsis (yes *vs* no)	0.6	−0.7; 2.1	0.30
Neurosensory diseases (yes *vs* no)	3.1	0.2; 3.8	0.06

PMA: postmenstrual age; CI: confidence interval; BPD: bronchopulmonary dysplasia; GI: gastrointestinal; PDA: patent ductus arteriosus.
